# Delphi expert consensus on phlebotomy limitations and treatment challenges in *HFE*-related hemochromatosis

**DOI:** 10.46989/001c.166769

**Published:** 2026-08-26

**Authors:** Jeremy Shearman, Domenico Girelli, Dominik J. Schaer, Sant-Rayn Pasricha, Kris V. Kowdley, John K. Olynyk, Gregory J. Kato, Sonya Abraham, Soraya Benchikh El Fegoun, Charlotte Pollet, Uta Merle

**Affiliations:** 1 Department of Gastroenterology, South Warwickshire University NHS Foundation Trust and Warwick Medical School, Warwick, UK; 2 Department of Medicine, Section of Internal Medicine and EuroBloodNet Referral Center, University of Verona and Azienda Ospedaliera Universitaria Integrata of Verona, Verona, Italy; 3 Department of Internal Medicine, University Hospital and University of Zürich, Zürich, Switzerland; 4 Population Health and Immunity Division, Walter and Eliza Hall Institute of Medical Research (WEHI), Melbourne, Australia; 5 Liver Institute Northwest, Seattle, Washington, USA; 6 Elson S. Floyd College of Medicine, Washington State University, Seattle, Washington, USA; 7 Curtin Medical Research Institute, Curtin University, Bentley, Western Australia, Australia; 8 Department of Gastroenterology, Fiona Stanley Hospital, Murdoch, Western Australia, Australia; 9 CSL Behring, King of Prussia, PA, USA; 10 CSL Behring, Bern, Switzerland; 11 CSL Vifor, Zürich, Switzerland; 12 Department of Internal Medicine IV, Gastroenterology & Hepatology, Medical University of Heidelberg, Heidelberg, Germany

**Keywords:** Delphi Consensus, Burden of Treatment, Phlebotomy, *HFE*-related Hemochromatosis

## Abstract

*HFE*-related hemochromatosis (HC) is caused by hepcidin dysregulation and is characterized by excessive iron absorption and accumulation in the liver, heart, and endocrine glands, which leads to complications including arthropathy, cirrhosis, and hepatocellular carcinoma. Standard of care (SoC) typically involves phlebotomy, with iron chelators used less frequently. However, these therapies may cause side effects and negatively impact quality of life, making them troublesome for some patients. Using the Delphi methodology, a survey was developed to identify unmet needs and clinical challenges within the current therapeutic landscape of HC, and to establish consensus statements for its management. Consensus was defined as ≥75% agreement. Thirty-two HC specialists from Europe, Australia and the USA responded to the survey. After three rounds of survey refinement, final consensus statements were compiled. The Delphi process identified key unmet needs in HC, including lack of alternatives to phlebotomy, persistent symptoms, and burden related to the use of current SoC. The consensus process helped establish definitions for high phlebotomy treatment burden, intolerance, and suboptimal response to phlebotomy. The process also identified the subgroup of patients overly burdened by phlebotomy. Patient-reported outcomes were considered key to assessing phlebotomy’s impact, although they are rarely measured in clinical practice. The Delphi study highlighted the limitations of phlebotomy, with respondents identifying a high unmet need for patients who cannot be managed with or do not tolerate this approach. The study suggested exploring alternative therapy options for patients who experience high treatment burden or intolerance to phlebotomy.

## 1. INTRODUCTION

Hemochromatosis is a genetic iron overload disorder marked by excessive iron absorption and overload in the joints and organs such as the liver, heart, and endocrine glands.^1^ The iron dysregulation is caused by low hepcidin levels or a reduced hepcidin-ferroportin binding interaction.^1,2^ This typically leads to increased transferrin saturation (TSAT) and the formation of non-transferrin-bound iron (NTBI). Progressive iron accumulation can then lead to oxidative stress and organ damage.^3^ The most common form of hemochromatosis is *HFE*-related hemochromatosis (HC), caused by homozygosity for the C282Y variant in the *HFE* gene encoding the hemochromatosis protein HFE, which regulates hepcidin expression.^3,4^ More than 80% of HC patients of Northern European descent are homozygous for this variant, reflecting its high prevalence within this population group.^3,5,6^ If left untreated, iron overload can lead to serious long-term complications, such as liver fibrosis, cirrhosis and hepatocellular carcinoma (HCC), heart failure, arthropathy, and diabetes.^7,8^

Standard of care for patients with HC consists of reduction in body iron stores by repeated phlebotomies (also known as therapeutic venesection). This technique of bloodletting dates back to ancient civilizations.^9^ First identified as a potential treatment for HC in the 1950s, phlebotomy is now considered the mainstay therapy for HC due to its simplicity and relatively low risks.^9^ Phlebotomy is generally well tolerated by a large proportion of patients, although it may be associated with side effects such as pain, hematoma, hypovolemia, and fatigue.^9^ Phlebotomy has been shown in several studies to improve survival,^9^ reverse liver fibrosis,^10–12^ lower the risk of HCC,^10^ and enhance cardiac function^13^ in patients with HC. The underlying mechanism by which iron overload contributes to joint damage remains unclear.^14^ This is supported by evidence from studies indicating that phlebotomy may only partially improve arthralgia or not improve it at all, and may fail to halt the progression of joint damage.^9,14–16^ Moreover, some patients may experience worsening joint symptoms following treatment.^15,16^ Notably, arthralgia and fatigue are the most commonly reported symptoms experienced by patients with HC who are referred for phlebotomy treatment.^17–19^

Newly diagnosed patients initially undergo frequent phlebotomies (generally weekly) during the ‘induction phase’ of therapy, with the aim of removing the excess iron stored in the body until a specific target serum ferritin level is reached. Patients will then be moved to the ‘maintenance phase’, which consists of less frequent phlebotomies to prevent iron re-accumulation.^9^ The 2022 European Association for the Study of the Liver (EASL) guidelines recommend a treatment target of <50 µg/L and 50–100 µg/L for serum ferritin levels in the induction and maintenance phases, respectively.^7^ No formal guidance on TSAT targets has been recommended due to the lack of high-quality evidence.^7,9,20^

Alternative treatments, used less frequently, include iron chelators and erythrocytapheresis.^9^ Although not currently approved for HC treatment, but indicated for iron overload due to blood transfusions or non-transfusion-dependent thalassemia, the iron chelator deferasirox has been investigated in patients with HC.^9,21,22^ Dose-dependent hepatic, renal, and gastrointestinal toxicities have been shown to be associated with its use.^9^ Iron chelators may be used in rare and exceptional circumstances such as juvenile hemochromatosis with cardiac dysfunction; this should be performed under close monitoring and at doses lower than 10 mg/kg/day.^9,22^

Erythrocytapheresis is a type of automated red blood cell exchange that has been shown to efficiently deplete iron while maintaining blood volume. Although it can remove 2.3 times the amount of iron compared with one phlebotomy session, the higher costs and unavailability in some healthcare settings impact on its widespread use.^9,22^

Although phlebotomy is the mainstay of therapy for patients with HC, procedure-related side effects are not infrequent, and its impact on a patient’s quality of life may be significant.^17^ In addition, a steady year-on-year decline in treatment compliance has been reported in patients undergoing maintenance therapy.^23^ It should also be noted that some patients with HC experience significant problems with phlebotomy treatment, and, as such, alternative treatment options may potentially benefit this population.^1,6,9^

This report presents the findings from a global Delphi consensus study involving HC specialists, aimed at identifying unmet clinical needs, burden of phlebotomy treatment, intolerance, and suboptimal response to phlebotomy, while highlighting opportunities to improve patient management and identifying alternative therapies, especially for the subgroup of patients who are intolerant or overly burdened by first-line phlebotomy treatment. The study focused exclusively on *HFE*-related hemochromatosis; here the abbreviation HC refers only to this form and does not encompass non-*HFE*-related hemochromatosis or secondary iron overload.

## 2. METHODS

### 2.1. Delphi methodology

The Delphi methodology^24,25^ was selected as a framework to achieve a consensus on the management of patients with HC among specialists in the field. A steering committee composed of six clinicians with direct experience and interest in managing HC developed the survey in line with the current body of evidence and expert understanding in the field. Following two online meetings, the survey was finalized by the steering committee. The survey was designed to obtain consensus on the following topics: unmet therapeutic needs, symptoms, high burden of treatment in the induction phase, high burden of treatment in the maintenance phase, phlebotomy intolerance, the impact of phlebotomy on a patient’s life, definition of phlebotomy treatment success and of failure to achieve target ferritin levels despite phlebotomy therapy, alternative therapies, effects of phlebotomy and long-term complications, and routine monitoring.

Consensus was determined based on the proportion of responses that agreed with a given statement, or the percentage of participants selecting a given answer from a multiple-choice question. The strength of consensus was defined as: strong consensus (≥75% agreement), moderate consensus (70–<75% agreement), partial consensus/majority approval (65–<70% agreement), and no consensus (<65% agreement). After each survey round, the data were processed and the strength of consensus was assessed. Statements achieving strong and moderate consensus were excluded from the subsequent survey round. Questions and statements from Round 1 and Round 2 surveys that obtained partial consensus or no consensus were refined by the steering committee to seek consensus in subsequent rounds. After three survey rounds, all statements achieving consensus were consolidated. A few statements failed to reach consensus following the three Delphi rounds and are detailed in this publication. Those experts who could not submit their responses in the first round but were interested in contributing to the following rounds, were still eligible to complete the survey distributed in Rounds 2 or 3. Steering committee members were also required to submit their responses.

For each round, the survey was programmed on the online platform SurveyMonkey®. A link to the survey was distributed to participants via email. The survey round was considered complete once a minimum of 30 participants had entered their responses.

For participant recruitment, the steering committee nominated several international HC specialists, who were then invited to participate in the process. Steering committee members also approved the inclusion of additional renowned experts in the field of HC identified by CSL Behring. Participation in the study was entirely voluntary, with no compensation provided to respondents for their input. All responses were anonymized.

### 2.2. Statistics

For each survey round, the consensus for each statement was determined by calculating the percentage of participants who agreed with it.

## 3. RESULTS

### 3.1. Panel characteristics

Between April 2025 and June 2025, 37 experts with a deep understanding of HC and its management were invited to participate in the Delphi study (Table 1). Overall, the majority of participants were from the United Kingdom (n=11; 29.7%), Australia (n=7; 18.9%), and Italy (n=6; 16.2%). Hepatology was the specialty with the highest representation (n=13; 35.1%) followed by internal medicine (n=7; 18.9%) and hematology (n=6; 16.2%).

**Table 1.** Details of participants in the Delphi consensus study, including steering committee members and recruited expert panel.

**Delphi participants (N=37)**	
**Country, n (%)**	
United Kingdom	11 (29.7)
Australia	7 (18.9)
Italy	6 (16.2)
United States	3 (8.1)
France	2 (5.4)
Germany	2 (5.4)
Spain	2 (5.4)
Austria	1 (2.7)
Belgium	1 (2.7)
Canada	1 (2.7)
Switzerland	1 (2.7)
**Medical specialty, n (%)**	
Hepatology	13 (35.1)
Internal medicine	7 (18.9)
Hematology	6 (16.2)
Gastroenterology	4 (10.8)
Genetics	4 (10.8)
Oncology	2 (5.4)
Rheumatology	1 (2.7)

### 3.2. Survey round refinement

**Figure 1.**
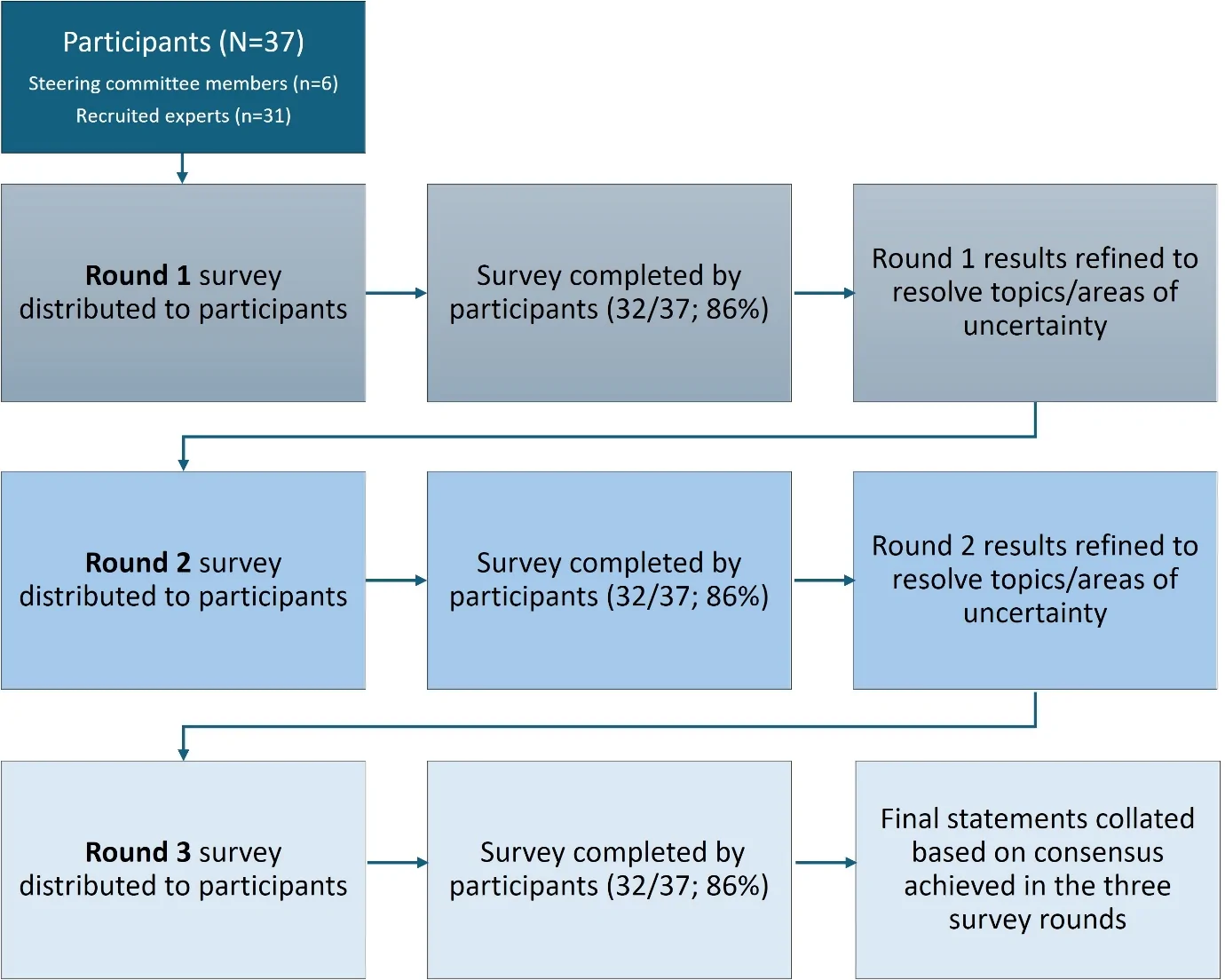
Flowchart illustrating the Delphi consensus process

The Delphi process and the number of participants who responded across the three survey rounds are illustrated in Figure 1. For each round, a total of 32 participants submitted their responses. Following Round 1, 13/31 questions did not reach consensus, whereas 7/31 received partial agreement. Respondents were in agreement on topics related to factors determining high burden of treatment in the induction phase, long-term complications of phlebotomy, definition of intolerance, and definition of treatment success. The main areas of disagreement were the specific phlebotomy frequency that defines a high burden of treatment in the induction and maintenance phases, the concept of treatment ‘refractoriness’, the use of magnetic resonance liver iron concentration (MRLIC), and the length of the monitoring intervals to assess organ damage. Round 1 contained mostly multiple-choice questions and free-text answers that were subsequently analyzed and revised/converted into statements as part of the second survey round. In Round 2, 6/35 questions remained unresolved due to a lack of consensus on the specific frequency of phlebotomy sessions that constitute a high burden of treatment in the maintenance phase, as well as the definition of failure to achieve target serum ferritin levels despite standard therapeutic phlebotomy. Unresolved questions related to specific monitoring intervals to assess organ function/damage and the impact of phlebotomy on stabilization/improvement of organ function were not included in Round 3. As such, the Round 3 survey included only four questions, mainly aimed at reaching consensus on defining high burden in the maintenance phase and the inability to achieve target serum ferritin levels following phlebotomy therapy. After the three rounds, results were collated and the steering committee proceeded to develop and finalize the statements.

### 3.3. Summary of consensus statements

Table 2 presents the 27 final statements compiled from the three survey rounds, organized under the 11 topics of the Delphi study. The following section includes the main findings of the study.

**Table 2.** Consensus statements for the management of patients with *HFE*-related hemochromatosis (HC).

**Statement**	**Level of agreement and strength of consensus**
**Topic 1. Unmet therapeutic needs**	
**1.1** Lack of alternative therapies to phlebotomy, symptoms that fail to resolve with current therapies, and insufficient understanding of how transferrin saturation and non-transferrin bound iron affect patient outcomes are the main unmet needs in HC	*Lack of alternative therapies* (**96.9%** strong consensus) *Symptoms that fail to resolve with current therapy* (**78.1%** strong consensus) *Insufficient understanding of how transferrin saturation and non-transferrin bound iron affect patient outcomes* (**100%** strong consensus)
**Topic 2. Persistent symptoms**	
**2.1** Arthralgia and fatigue are symptoms in HC that are not currently addressed with available therapies	*Arthralgia* (**84.4%** strong consensus) *Fatigue* (**96.9%** strong consensus)
**Topic 3. High burden of treatment in induction phase**	
**3.1** Among HC healthcare professionals, it is recognized that some patients experience high burden of phlebotomy treatment	**100%** strong consensus
**3.2** In the initial/induction phase, frequency of phlebotomy sessions (i.e., ≥3–4 times a month), the impact on daily activities (e.g., time off work, travel, waiting times, childcare), and severity of treatment-related adverse events (e.g., fatigue, dizziness) are the most indicative factors of high treatment burden	*Frequency of phlebotomy sessions* (**93.8%** strong consensus) *Impact on daily activities* (**81.3%** strong consensus) *Severity of treatment-related adverse events* (**71.9%** moderate consensus)
**3.3** In the initial/induction phase, a phlebotomy frequency of once a week or more frequently, is considered a high burden of treatment	**81.3%** strong consensus
**3.4** Frequency and duration of phlebotomy treatment during the induction phase are considered major limitations of phlebotomy	**75.0%** strong consensus
**3.5** It is expected that a patient with a baseline ferritin serum level 1,000–2,500 μg/L may spend 9–18 months in the induction phase before moving to maintenance treatment	**87.1%** strong consensus^a^
**Topic 4. High burden of treatment in maintenance phase**	
**4.1** The severity of treatment-related adverse events (e.g., fatigue, dizziness) is considered the most important issue faced by patients during the maintenance phase	**75.0%** strong consensus
**4.2** Impact on daily activities (e.g., time off work, travel, waiting times, childcare) are indicative of high burden of treatment	**68.8%** partial consensus/majority approval
**4.3** In the maintenance phase, ≥6 phlebotomies/year are considered the minimum number of sessions constituting high burden	**59.4%** no consensus^b^
**Topic 5. Phlebotomy intolerance**	
**5.1** It is widely recognized that some patients are intolerant to phlebotomy	**100%** strong consensus
**5.2** Intolerance to phlebotomy refers to the patient's inability to tolerate phlebotomy treatment due to occurrence of adverse effects, comorbidities, or exacerbation of existing medical conditions	**90.6%** strong consensus
**5.3** Difficult venous access, unpleasant reactions (e.g., vasovagal), and needle aversion are frequently cited as reasons patients are unable to continue with phlebotomy therapy	*Difficult venous access* (**78.1%** strong consensus) *Unpleasant reactions* (**71.9%** moderate consensus) *Needle aversion* (**81.3%** strong consensus)
**Topic 6. Impact of phlebotomy on a patient’s life**	
**6.1** Patient-reported outcomes are of highest importance to patients when assessing the impact of phlebotomy on their life; however, this impact is not commonly measured in clinical practice	*Patient-reported outcomes* (**87.5%** strong consensus) *Impact not being commonly measured in clinical practice* (**81.3%** strong consensus)
**Topic 7. Definition of phlebotomy treatment success**	
**7.1** Phlebotomy treatment success may be defined as improvement in symptoms and overall quality of life, imaging-based reduction in tissue iron concentration, and reduction in iron-mediated end-organ damage	*Improvement in symptoms* (**71.0%** moderate consensus)^a^ *Improvement in overall quality of life* (**74.2%** moderate consensus)^a^ *Imaging-based reduction in tissue iron concentration* (**74.2%** moderate consensus)^a^ *Reduction in iron-mediated end-organ damage* (**83.9%** strong consensus)^a^
**Topic 8. Definition of failing to achieve target ferritin levels despite phlebotomy therapy**	
**8.1** A failure to achieve treatment target despite standard therapeutic phlebotomy is defined as insufficient or suboptimal response	**93.8%** strong consensus
**Topic 9. Alternative therapies**	
**9.1** If phlebotomy is deemed no longer appropriate for managing a patient, it is acceptable to move to next line treatment within a month after the last phlebotomy session	**84.4%** strong consensus
**9.2** Failure to achieve treatment target outcomes is typically defined after a minimum duration of 6 months	**83.9%** strong consensus^a^
**9.3** Hepcidin-ferroportin-targeted therapies alone or combined with phlebotomy currently represent the most promising treatment options for patients with HC	**87.5%** strong consensus
**9.4** Patients experiencing high burden of treatment and intolerance to phlebotomy should be offered second-line or alternative treatments	*High burden of treatment* (**87.5%** strong consensus) *Intolerance to phlebotomy* (**86.7%** strong consensus)^c^
**Topic 10. Effects of phlebotomy and long-term complications**	
**10.1** In patients with HC, phlebotomy treatment is expected to: Improve liver function tests Stabilize skeletal complications Improve or stabilize cardiac dysfunction and arrhythmias as assessed via electrocardiogram (ECG) and echocardiography	*Improve liver function tests* (**100%** strong consensus)^a^ *Stabilize skeletal complications* (**74.2%** moderate consensus)^a^ *Improve or stabilize cardiac dysfunction and arrhythmias as assessed via ECG and echocardiography* (**84.4%** strong consensus)
**10.2** Venipuncture site issues are considered long-term complications associated with repeated phlebotomies	**80.0%** strong consensus
**Topic 11. Routine monitoring**	
**11.1** In patients with HC, regular monitoring in the maintenance phase should include: Ferritin serum level assessments Liver function tests Diabetes screening Joint assessments (every 12 months or longer intervals) Ultrasound for hepatocellular carcinoma surveillance in patients with significant liver disease (every 6 months)	*Ferritin serum level assessments* (**96.7%** strong consensus) *Liver function tests* (**71.9%** moderate consensus) *Diabetes screening* (**71.9%** moderate consensus) *Joint assessments* (**78.1%** strong consensus) *Every 12 months or longer intervals* (**88.0%** strong consensus)^d^ *Ultrasound for hepatocellular carcinoma surveillance in patients with significant liver disease* (**100%** strong consensus) *Every 6 months* (**78.1%** strong consensus)
**11.2** Regular monitoring of pituitary hormone levels in patients with HC are not required; tests should be performed based on emergent symptoms	**78.1%** strong consensus
**11.3** Magnetic resonance imaging (MRI) should be considered a valuable non-invasive modality for the quantification of liver iron concentration in selected patients with HC; routine assessments may be performed at intervals of 12 months or longer	*Use of MRI as a valuable modality to quantify liver iron concentration in selected patients* (**96.7%** strong consensus) *Assessment performed at 12 months or longer intervals* (**93.6%** strong consensus)^e^
**11.4** Routine monitoring of transferrin saturation (TSAT) is considered clinically appropriate in the management of HC	**81.3%** strong consensus
**11.5** The ongoing management and routine clinical assessment of patients with HC should be overseen by a qualified specialist experienced in iron overload disorders (i.e., hematologist, hepatologist, gastroenterologist, internal medicine specialist)	**80.0%** strong consensus^c^

^a^One participant did not provide their response; overall percentage was calculated for the 31 participants providing a response for the specific question.

^b^21.9% of respondents considered high burden to be ≥5 phlebotomies/year.

^c^Two participants did not provide their responses; overall percentage was calculated for the 30 participants providing a response for the specific question.

^d^Overall, 22 of 25 respondents were in agreement to perform routine joint assessments every 12 months or longer; this included also a respondent who had answered not to agree with performing routine joint assessments.

^e^Level of consensus calculated as the percentage of the participants in agreement with the use MRI to measure liver iron concentration in selected patients (n=31).

Respondents agreed that the key unmet therapeutic needs in HC are the lack of alternative therapies to phlebotomy (96.9%), symptoms that do not resolve with current therapies (78.1%), and insufficient understanding of how TSAT and NTBI affect patient outcomes (100%). Respondents agreed that fatigue (96.9%) and arthralgia (84.4%) represent the main symptoms that can remain unresolved with first-line phlebotomy therapy.

All respondents agreed that some patients experience a high phlebotomy treatment burden during the initial/induction phase, mostly due to session frequency (93.8%), severity of treatment-related adverse events (71.9%), and impact on daily activities (81.3%). Frequency and duration of phlebotomy treatment during induction were identified as significant limitations by 75.0% of respondents, with a frequency of more than once a week identified as a high treatment burden for patients in this initial phase of therapy (81.3%). Respondents agreed that a patient with a baseline serum ferritin level 1,000–2,500 μg/L will typically spend a period of 9–18 months in the induction phase before moving to the maintenance phase (87.1%).

Following three rounds of survey, no consensus was reached with regard to the minimum number of sessions constituting a high burden of treatment for a patient in the maintenance phase. While the majority of respondents (59.4%) indicated ≥6 phlebotomies/year, 21.8% of respondents considered ≥5 phlebotomies/year a high treatment burden. The severity of treatment-related adverse events (e.g., fatigue, dizziness) was considered by the respondents to represent the greatest challenge faced by their patients during this phase (75.0%).

Overall, 90.6% of respondents agreed with the definition of intolerance to phlebotomy being a patient’s inability to tolerate phlebotomy treatment due to adverse effects, comorbidities, or exacerbation of existing medical conditions. Common reasons patients cite as feeling ‘unable to continue’ phlebotomy therapy include difficult venous access (78.1%), unpleasant reactions such as vasovagal episode or syncope (71.9%), and needle aversion (81.3%).

The experts agreed that patient-reported outcomes are of highest importance to patients when assessing the impact of phlebotomy on their lives (87.5%); however, they also concurred that this impact is not commonly measured in clinical practice (81.3%).

Following Round 2 of the survey, it was agreed that the concept of ‘treatment refractoriness’ is not appropriate to define the failure to achieve treatment target despite a course of therapeutic phlebotomy; instead, respondents concurred that the term ‘insufficient or suboptimal response’ should be used in the context of HC (93.8%).

Respondents (87.5%) agreed that hepcidin-ferroportin-targeted therapies with or without phlebotomy are the most promising HC treatment options and that patients experiencing high phlebotomy burden (87.5%) and intolerance (86.7%) should be offered a second-line or alternative therapy. The panel determined that a minimum period of 6 months is required before confirming a failure to achieve treatment target outcomes (83.9%). On the other hand, respondents considered phlebotomy treatment success to be an improvement in symptoms (71.0%) and overall quality of life (74.2%), an imaging-based reduction in tissue iron concentration (74.2%), and a reduction in iron-mediated end-organ damage (83.9%).

Overall, there was consensus on the effect/impact of phlebotomy on the key organs affected by iron accumulation (Table 2, Statement 10.1) and respondents considered venipuncture site issues as long-term complications from repeated phlebotomies (80.0%).

For optimal patient outcomes, respondents concurred that regular monitoring in the maintenance phase should include serum ferritin levels (96.7%), joint assessment (78.1%) every ≥12 months (88.0%), HCC surveillance in the presence of advanced liver disease (100%) every 6 months (78.1%), liver function biochemistry tests (71.9%), and diabetes mellitus assessment (71.9%). Additional monitoring of TSAT was also considered appropriate in clinical practice (81.3%). Almost all respondents (96.7%) agreed that magnetic resonance imaging (MRI) may be valuable for quantifying liver iron concentration in selected patients; measurements may be taken at intervals of 12 months or longer (93.6%). Respondents also concurred that the management and routine clinical assessments of patients with HC should be carried out by a qualified specialist medical practitioner experienced in the management of the condition (80.0%).

## 4. DISCUSSION

The findings of this study provide additional information on the clinical management of HC by identifying current unmet needs and unresolved symptoms, and defining a high burden of phlebotomy, treatment intolerance, and an insufficient or suboptimal response to first-line phlebotomy. The study also confirmed that clinicians acknowledge the fact that their patients experience phlebotomy-related adverse effects and report symptoms such as fatigue and arthralgia, which may not respond to phlebotomy treatment. Although phlebotomy is effective in most patients, in some individuals iron overload persists or ferritin targets are not achieved, and these individuals may therefore require a different therapeutic approach.^9^

One aim of the Delphi consensus was to establish a definition of high phlebotomy burden in patients with HC, as no standardized definition currently exists. Although consensus was not fully reached for the frequency of phlebotomies in the maintenance phase, the majority of respondents agreed that ≥1/week phlebotomies in the induction phase and ≥6/year in the maintenance phase represent a high burden for patients. It is important to consider that the question was posed to clinicians and experts in HC and, therefore, may differ from a definition provided by patients; nevertheless, this is the first study that aimed at addressing this gap within the literature. Defining burden of phlebotomy and quantifying its frequency may support healthcare providers in identifying patients who are intolerant to phlebotomy, have had an insufficient response to phlebotomy, or experience a high burden and who may benefit from alternative therapies. Although high treatment frequency may contribute to the overall burden of phlebotomy, it is not the primary burden driver for most patients. Instead, the burden is largely attributed to reported adverse effects (i.e., fatigue, dizziness) and the impact on daily activities, including disruptions to work, travel, and social life. As such, the perceived burden of treatment should be discussed with patients and assessed on an individual basis by the treating clinician. In fact, a proportion of patients may be dissatisfied with their treatment regime, the inconvenience of the procedure, and/or the frequency of side effects.^17^ Results from a survey of patients with HC investigating the impact of phlebotomy treatment on their quality of life, indicated that 16% of patients undergoing regular phlebotomies would ‘definitely’ or ‘probably’ choose not to receive phlebotomy if alternative therapies were available to them.^17^ Of note, this survey was conducted before the key discoveries on the role of the hepcidin-ferroportin axis in the pathophysiology of HC.^1^ Although there was an initial lack of consensus regarding phlebotomy frequency and its impact on daily life during maintenance, respondents agreed that the severity of adverse events is the most important issue faced by patients. Although not directly linked to severity, it is interesting to note that the study by Brissot et al.^17^ reported that 52% of induction and 37% of maintenance patients experienced adverse effects, such as tiredness, fainting, and loss of appetite, ‘always’ or ‘most of the time’ after phlebotomy, with the most frequently reported side effect being tiredness (58% of induction and 50% of maintenance patients).^17^ Other complications of phlebotomy include syncope/vasovagal reaction, hematoma, compartment syndrome, and calcification or scarring of the vein wall.^9^ Treatment inconvenience and the incidence of adverse events may also be responsible for the overall decline in treatment compliance during the maintenance phase.^23^ In a large survey of patients with HC from the United States and Canada, the key negative aspects of phlebotomy that were identified included venous access issues, time for travel and to carry out the procedure, and the fact that, in many cases, the blood cannot be used for donation.^18^ Fifty-nine percent of respondents indicated that they would opt for a pill with a 5% risk of serious adverse effects over phlebotomy.^18^ This is in line with the findings from the current consensus work, additionally confirming that from the experts’ perspectives, the impact of phlebotomy on a patient’s life should be assessed by measuring patient-reported outcomes; however, they also acknowledged that this type of assessment is not commonly performed in clinical practice, highlighting an important gap in a patient’s clinical management. A previously published patient survey revealed that both the frequency and duration of phlebotomy sessions were perceived as burdensome, with phlebotomy considered inconvenient by 29% of patients in the induction phase and 25% in the maintenance phase.^17^ Furthermore, 34% reported that phlebotomy limited their daytime activities ‘always’ or ‘most of the time’, and 25% indicated a reliance on others due to treatment.^17^ These findings highlight the significant impact of phlebotomy on patients’ daily lives and underscore the need for more patient-centric approaches to iron depletion therapy.

Consistent with the literature,^17–19^ our study identified fatigue and arthralgia as symptoms of HC which are common and may persist despite phlebotomy therapy. While some improvement in fatigue has been observed following use of erythrocytapheresis,^26^ it remains unclear why arthralgia and arthropathy may worsen following phlebotomy therapy.^9,14–16,27^ Concerns about arthritis and joint issues have also been emphasized in a survey of patients with HC.^28^ When asked to identify research priorities, 45.3% of respondents ranked arthritis and joint problems as the top areas for future research in the HC field.^28^ In addition, due to the associated arthropathy, patients with HC face a significantly increased risk of joint replacement surgery,^29,30^ being nine times more likely to undergo the procedure compared with the general population.^31^

Among experts, a strong consensus (90.6%) was reached in defining intolerance to phlebotomy as the patient’s inability to tolerate treatment due to the occurrence of adverse effects, comorbidities, or exacerbation of existing medical conditions. Although phlebotomy is generally well tolerated and highly effective, it may not be feasible at the required frequency in patients with poor tolerance, such as those with inaccessible veins, needle aversion, concomitant anemia, or medical conditions where the procedure could cause harm.^7^ As revealed from the findings of this Delphi process, participants confirmed that patients often cite difficult venous access, needle aversion, and unpleasant reactions (i.e., vasovagal episodes) as reasons to discontinue phlebotomy. This is consistent with the survey by Brissot et al. that reported that 22% of patients were ‘quite bothered’ or ‘very bothered’ by the use of a needle for phlebotomy, and 30% of induction patients and 20% of maintenance patients experienced problems with needles ‘most of the time’ or ‘always’.^17^

As opposed to phlebotomy intolerance, the concept of ‘treatment refractoriness’ proved much more controversial among participants. As a result of the Delphi process, experts concurred that the term ‘insufficient or suboptimal response’ to phlebotomy should be used to describe the failure to achieve the treatment target despite therapeutic phlebotomy. For this specific group of patients, the American College of Gastroenterology (ACG) guidelines recommend the use of iron chelators as an alternative treatment.^16^ This recommendation was based on the positive results of the Phase 2 study of the iron chelator deferasirox,^32^ although the study was terminated more recently due to difficulty in patient enrollment.^22,33^ Advocated by clinical experts,^9^ research on alternative therapies represents one of the top priorities also identified by patients.^28^ Interestingly, the Delphi panel indicated hepcidin-ferroportin-targeted therapies alone or in combination with phlebotomy as the most promising therapies to date. A recent proof-of-concept Phase 2 study has shown that the hepcidin mimetic rusfertide (Takeda) can prevent iron re-accumulation in the absence of phlebotomies and may represent a viable therapeutic option for selected patients with HC.^34^ Another attractive molecular-targeted therapy may also be represented by vamifeport (CSL), a small molecule and selective ferroportin inhibitor that acts as a hepcidin mimetic,^35^ whose preclinical proof-of-concept study suggested its potential in HC management.^2^ In addition, its oral route of administration may also enable a reduction in treatment burden for patients. However, large clinical trials are needed to confirm the effectiveness of these therapies and/or their roles in clinical management.^9^

As part of the regular monitoring of patients in the maintenance phase, respondents indicated that ferritin levels, joint assessment, HCC surveillance in patients with advanced liver disease, liver function tests, and diabetes should be assessed. These recommendations are in line with current clinical guidelines.^7^ Monitoring of TSAT was also considered appropriate in clinical practice by 81.3% of respondents; however, specific targets for TSAT (<50–60%) have only been provided by the British Society for Haematology (BSH) and the Danish national guidelines.^7,20,36^ Such targets are supported by the evidence from a study showing that patients who failed to maintain TSAT <50% might be at increased risk of developing arthropathy typically associated with HC.^36,37^

Delphi respondents agreed that the use of MRI, if available, may be useful for estimating liver iron levels in selected patients. MRI has proven valuable for assessing liver iron burden, guiding clinical decisions, and ruling out significant iron overload, particularly in individuals with marked hyperferritinemia and ‘low-risk’ *HFE* genotypes, such as those without C282Y homozygosity.^9,38–40^

A key strength of this study lies in the use of the Delphi methodology, which enabled consensus among a large, geographically diverse panel of experts in HC from Europe, Australia, and the United States. The high retention rate throughout the survey rounds contributed to robust agreement on several topics highly relevant to the clinical management of HC. The structured, iterative nature of the Delphi process also facilitated the identification of shared priorities and unmet needs, while minimizing bias and promoting balanced expert input.

A potential limitation of this study is that, although healthcare professionals are well qualified to assess the clinical tolerability and adequacy of response to phlebotomy, a comprehensive evaluation of treatment burden in HC may require alternative research approaches that take into account patient perspectives and patient-reported outcomes rather than clinician perspectives and clinical assessments.^41^

Overall, we anticipate that this consensus statement will optimize the management of patients with HC and may aid healthcare systems and patients in their decision-making process.

## 5. CONCLUSION

The Delphi consensus survey highlighted several unmet needs in the management of patients with HC, particularly the high burden of phlebotomy treatment and its limitations especially in controlling common symptoms like fatigue and arthralgia. Respondents indicated a substantial unmet need among patients who experience high treatment burden or who poorly tolerate phlebotomy and identified molecular-targeted therapies that address the pathophysiological root cause of HC as a suitable therapeutic option for this subgroup of patients.

### Ethics approval

Not applicable

### Consent to participate

All participants provided informed consent to take part in the Delphi study prior to participation.

### Consent for publication

Not applicable

### Author contributions

Conceptualization: Jeremy Shearman (Equal), Domenico Girelli (Equal), Dominik J. Schaer (Equal), Sant-Rayn Pasricha (Equal), Kris V. Kowdley (Equal), Uta Merle (Equal). Methodology: Jeremy Shearman (Equal), Domenico Girelli (Equal), Dominik J. Schaer (Equal), Sant-Rayn Pasricha (Equal), Kris V. Kowdley (Equal), Soraya Benchikh El Fegoun (Equal), Uta Merle (Equal). Data curation: Jeremy Shearman (Equal), Domenico Girelli (Equal), Dominik J. Schaer (Equal), Sant-Rayn Pasricha (Equal), Kris V. Kowdley (Equal), John K. Olynyk (Equal), Gregory J. Kato (Equal), Sonya Abraham (Equal), Charlotte Pollet (Equal), Uta Merle (Equal). Formal Analysis: Jeremy Shearman (Equal), Domenico Girelli (Equal), Dominik J. Schaer (Equal), Sant-Rayn Pasricha (Equal), Kris V. Kowdley (Equal), John K. Olynyk (Equal), Gregory J. Kato (Equal), Sonya Abraham (Equal), Charlotte Pollet (Equal), Uta Merle (Equal). Writing – review & editing: Jeremy Shearman (Equal), Domenico Girelli (Equal), Dominik J. Schaer (Equal), Sant-Rayn Pasricha (Equal), Kris V. Kowdley (Equal), John K. Olynyk (Equal), Gregory J. Kato (Equal), Sonya Abraham (Equal), Soraya Benchikh El Fegoun (Equal), Charlotte Pollet (Equal), Uta Merle (Equal). Project administration: Soraya Benchikh El Fegoun (Equal), Charlotte Pollet (Equal).

### Competing interests

JS received honoraria for participating in the steering committee for the Delphi survey and consulting/advisory fees from CSL Behring. DG has participated on advisory boards for Sanofi, Kedrion-Pharmacosmos, Vifor Pharma, and Novo Nordisk, and received honoraria for participating in the steering committee for the Delphi survey. DJS received honoraria for participating in the steering committee for the Delphi survey. SP holds a patent and receives royalties from Silence Therapeutics, holds an unremunerated role as Director of the WHO Collaborating Centre for Anaemia Detection and Control, has received an unrestricted research grant as a co-investigator from Vifor Pharma Ltd and received honoraria from CSL Behring for participating in the steering committee for the Delphi survey. KVK has received research support from CymaBay; grants and/or contracts from 89Bio, Genfit, Gilead, GSK, Hanmi, HighTide, Intercept, Madrigal, Mirum, NGM, Pfizer, Pliant, and Viking; royalties/licenses from UpToDate; consulting fees from 89Bio, Calliditas, CymaBay, Genfit, Gilead, Inipharm, Intercept, Madrigal, Mirum, NGM, and Pliant; payment/honoraria for lectures, presentations, speaker bureaus, manuscript writing, or educational events from AbbVie, Gilead, and Intercept; payment for expert testimony from the Department of Justice. He has also participated in a data safety monitoring board or advisory board for CTI and Medpace, has stock or options in Inipharm, and has received equipment from Sonic Incytes; he also received honoraria for participating in the steering committee for the Delphi survey and consulting/advisory fees from CSL Behring. JKO received consulting/advisory fees from CSL Behring. GJK, SA, and SBEF are employees of CSL Behring. CP is an employee of CSL Vifor. UM has received speaker and consultancy honoraria from Boehringer Ingelheim, CytoSorbents, Falk Foundation, Gilead, GSK, Ipsen, Microbiotica, and Univar; she has received travel fees from Gilead; and she received honoraria for participating in the steering committee for the Delphi survey and consulting/advisory fees from CSL Behring.

## Data Availability

Data are available from the authors upon reasonable request.
